# Sex-specific *dmrt1* and *cyp19a1* methylation and alternative splicing in gonads of the protandrous hermaphrodite barramundi

**DOI:** 10.1371/journal.pone.0204182

**Published:** 2018-09-18

**Authors:** Jose A. Domingos, Alyssa M. Budd, Quyen Q. Banh, Julie A. Goldsbury, Kyall R. Zenger, Dean R. Jerry

**Affiliations:** 1 Tropical Futures Institute, James Cook University Singapore, Singapore; 2 Centre for Sustainable Tropical Fisheries and Aquaculture, College of Science and Engineering, James Cook University, Townsville, Queensland, Australia; University of Hyderabad, INDIA

## Abstract

Epigenetics is involved in sex differentiation of gonochoristic and hermaphroditic fish species, whereby two genes *dmrt1* (pro-male) and *cyp19a1* (pro-female) are known to play major roles. Barramundi, *Lates calcarifer*, is an important tropical aquaculture species that undergo natural and permanent male to female sex change, a process for which the exact underlying molecular mechanisms are still unknown. To elucidate whether DNA methylation is involved in sex control of barramundi, a next-generation bisulfite amplicon sequencing approach was used to target 146 CpG sites within proximal promoters and first exons of seven sex-related genes (*dmrt1*, *cyp19a1*, *amh*, *foxl2*, *nr5a2*, *sox8* and *sox9*) of 24 testis and 18 ovaries of captive and wild adult barramundi. Moreover, comparative expression profiles of the key *dmrt1* and *cyp19a1* genes were further investigated using RT-qPCR and Sanger sequencing approaches, whereas expression levels of remaining targeted genes were based on available literature for the species. Results showed that *cyp19a1* and *amh* were more methylated in males, whereas *dmrt1* and *nr5a2* were more methylated in females (*P* < 0.001), with no gender differences found for *foxl2*, *sox8* or *sox9* genes (*P* > 0.05). Sex-biased promoter DNA methylation was inversely related to gene expression only for *dmrt1* and *nr5a2*, and directly related to *amh* expression, whereas no differences in *cyp19a1* expression were found between testes and ovaries. Notably, unique sex-specific alternative splicing of *dmrt1* and *cyp19a1* were discovered, whereby males lacked the full-length aromatase coding *cyp19a1* mRNA due to partial or total exon splicing, and females lacked the *dmrt1* exon containing the DM-domain sequence. This study advances the current knowledge aiming to elucidate the genetic mechanisms within male and female gonads of this large protandrous hermaphrodite by providing the first evidence of epigenetics and alternative splicing simultaneously affecting key genes (*cyp19a1* and *dmrt1*) central to sex differentiation pathways.

## Introduction

Teleosts display a variety of sex determination mechanisms incorporating biochemical steps whose regulation depends on genetic switches, environmental factors, or occasionally on their interaction [1, 2]. Such mechanisms are involved with gonadal differentiation itself, which in fish occurs in various forms from gonochoristic species (individuals directly develop and possess either testes or ovaries throughout their lifetime), simultaneous hermaphrodites (individuals possess both testes and ovaries), protogynous hermaphrodites (initial gonad development of ovaries with subsequent sex-change to testes), or protandrous hermaphrodites (initial gonad development of testes with subsequent sex-change to ovaries). In fact, teleost fish are the only vertebrate group known to undergo natural sex change; however, the underlying molecular mechanisms allowing such physiological and morphological changes that transform one sex into the other still remains poorly understood [3, 4]. In a small number of gonochoristic species, the master switches controlling primary sex determination have been identified (e.g. *amhy* in Nile tilapia *Oreochromis niloticus* [5]). In sequential hermaphrodites, which lack any form of sex-chromosomes [6], gonadal fate is labile throughout ontogeny and supposedly determined by environmental and/or endocrine forces that can tip the balance towards one or the other sex [7]. For hermaphrodites, it is possible that alternative molecular mechanisms, such as epigenetic switches, may not only be activating a masculinising or feminising network of several downstream sex-differentiating genes which orchestrate gonadal differentiation and maintenance, but may also be suppressing the other antagonistic sex network [7].

Exposure to temperature changes during early larval stages can induce epigenetic modifications, such as DNA methylation [8–11]. This DNA methylation often affects gene expression, sex differentiation and expected sex ratios of a number of fish species; even those species where sex is primarily controlled by multiple genes (e.g. European seabass *Dicentrarchus labrax*) [8, 12], or a single master gene (e.g. Nile tilapia) [13, 14]. The majority of work in plasticity of sex determination show differential DNA methylation levels within the proximal promoter and first exon of two major sex-related genes, *cyp19a1* (cytochrome P450, family 19, subfamily a, responsible for the aromatization of androgens into estrogens) and/or *dmrt1* (doublesex and mab3 related transcription factor 1, associated with male differentiation), although genome-wide differentially methylated regions have been observed between teleost testes and ovaries [14, 15]. For instance, in the gonochoristic Japanese flounder (*Paralichtys olivaceus*) DNA methylation of *dmrt1* and *cyp19a1* promoters were inversely correlated with *dmrt1* and *cyp19a1* transcription in an opposing manner between male and female gonads [11]. However, new evidence suggests that epigenetic mechanisms may also underlie sex determination and differentiation in sequential hermaphrodite fish. For example, in the protogynous ricefield eel (*Monopterus albus*), methylation of the *cyp19a1* promoter increases as individuals approach sex change, with an associated decline in transcription rate at the time of ovarian to testicular tissue change [16]. More recently, Wu et al. [17] demonstrated the inverse phenomenon in the digonic, protandrous black porgy (*Acanthopagrus schlegelii*), a species with functional testis and non-functional ovarian tissue in the first two reproductive seasons, which then sex change to functional females in the third reproductive season. In the black porgy, the *cyp19a1* promoter in ovarian tissue became progressively demethylated as the individuals develop into functional females, corresponding to higher *cyp19a1* transcriptional levels as the ovaries became fully functional [17]. In a recent large-scale transcriptomic study on the hermaphrodite clownfish (*Amphiprion bicinctus*), Casas et al. [18] have shown that the genetic mechanism underlying sex change in that species is strongly linked with the sex steroidogenic machinery, whereby key sex determining genes, namely *dmrt1*, *amh* and *sox8* operate as male-biased and *cyp19a1* and *foxl2* operate as female-biased genes. Whether gonadal DNA methylation status of such sex genes is also associated with the alternative gonadal phenotypes of hermaphrodite fishes, in synchronicity with *cyp19a1* as demonstrated in the protogynous ricefield eel [19] and in the bigonic protandrous black porgy [17], still remains unknown.

Barramundi, also known as Asian seabass (*Lates calcarifer*), is a large protandrous hermaphrodite of commercial importance for fisheries and aquaculture in Southeast Asia and Australia, with production increasingly globally [20]. In barramundi, testicular tissues develop from an undifferentiated gonad during the second to sixth month of age, with spermatozoa released in the efferent ducts as early as four months of age [21]. Males are fully mature at about 3–4 years and sex change into female between 4–8 years of age [21], although this change can occur as early as 2–3 years of age in captive situations [22]. Barramundi do not possess an ovotestis gonad, with the ovarian lumen forming *de novo* through profound morphological and histological changes in which testicular tissue degenerates within the solid lobes of the testes and ovarian tissue arises from ventral regions [22]. Such changes were also shown at the molecular level by the sexually dimorphic expression of several sex related-genes, leading to the suggestion that protandry in barramundi is likely to follow an inverse mechanism to that in the zebrafish (*Danio rerio*) ovary-to-testis transformation [23]. Although the aforementioned relationships among fish age and size, gonadal morphology, gene expression levels in adult barramundi have contributed to a general understanding of protandry in the species, and the availability of a well characterized genome [24], putative epigenetic factors operating within sex related-genes of barramundi male or female gonads are still unknown.

To investigate whether epigenetic mechanisms and transcription of the central sex genes *dmrt1* and *cyp19a1* are involved in the maintenance of the different sexual phenotypes in the protandrous barramundi, testicular and ovarian DNA methylation and expression levels from both captive broodstock and wild adult were assessed using bisulfite amplicon next-generation sequencing (BSAS), RT-qPCR and Sanger sequencing. In addition, the DNA methylation levels of an additional five genes implicated in sex differentiation (*sox8*, *sox9*, *foxl2*, *nr5a2* and *amh*) within male and female gonads were also investigated and results obtained discussed from data available in published gene expression datasets for the species [23].

## Materials and methods

### Ethics statement

This study was conducted in accordance with the Australian Code for the Care and Use of Animals for Scientific Purposes (National Health and Medical Research Council, 2013), in compliance with the Queensland Animal Care and Protection Act 2001 and James Cook University Animal Ethics Committee approval #A2014. Barramundi is not an endangered or protected species in Australia. Wild caught samples were purchased from commercial fishermen as part of regular fishing activities and outside of protected areas, whereby no special permits are needed.

#### Sample collection

The study was conducted on testes and ovaries of wild and captive *L*. *calcarifer* adults. Fourteen wild barramundi (10 males and four females) were collected via gill net in North Queensland (-17.221151° latitude; 145.984632° longitude), Australia. Nets were checked regularly and once caught, fish were euthanized in an ice slurry and gonads were immediately dissected. Of note, only four wild females out of 80 fish (~5% of total) were captured over six fishing expeditions and no transitional wild or captive broodstock fish were found throughout the study. Samples from captive adult barramundi (14 males and 14 females) were collected at the James Cook University/Mainstream Aquaculture Pty Ltd barramundi hatchery facility through cannulation biopsy of the gonads (BD Intramedic, Becton Dickinson) on anesthetized (AQUI-S New Zealand Ltd) broodstock during routine checking of fish sex and reproductive condition. Samples were preserved in RNAlater solution (Ambion), held at 4 °C overnight and then stored at -20 °C until DNA and RNA extraction. All 42 gonadal samples (24 testes and 18 ovaries) were subjected to BSAS, five to 10 samples of each gonadal type were subject to RT-qPCR and three testes and three ovaries of wild fish were subjected to confirmatory histological examination.

### Sex genes targeted for DNA methylation and gene expression

A suite of seven key genes of known sex differentiation and reproductive function in fish and vertebrates, *dmrt1*, *cyp19a1*, *foxl2*, *nr5a2*, *amh*, *sox8 and sox*9 previously characterised in barramundi [25] were targeted for the comparative assessment between the epigenetic profiles in *L*. *calcarifer* testes and ovaries. Putative transcription factor binding sites (TFBS) for the promoter regions were predicted using MatInspector software [26]. Differential DNA methylation patterns in a total of 146 CpG sites were assessed through BSAS [27]. BSAS allows the simultaneous processing of numerous different amplicons from hundreds of individually barcoded samples through small benchtop next-generation sequencers. Primers for bisulfite amplicon sequencing (Table 1) were designed around proximal promoter regions and first exons using MethPrimer [28]. Primer pairs were tested to produce single amplicons when bisulfite converted DNA was used and yield no amplification product when (unconverted) genomic DNA was used as template.

**Table 1:** *Lates calcarifer* sex-related genes and primer sequences investigated for bisulfite amplicon next-generation sequencing (BSAS) and gene expression.

Gene (amplicon number)	Accession	Primer name	Primer sequence (5’—3’) ^1^
*dmrt1 (1)*	KR232516.1	D1-BS-P-F1Seq	FO–GTTGATTAGGATTTGTGTTTTAAAGT
(BSAS)		D1-BS-P-R1Seq	RO–TAAAACCTATTATTTCATATAAACATATTT
*dmrt1 (2)*		D1-BS-P-F2Seq	FO–AAATTAAGTGTAGTAGAGTGATGTTAT
(BSAS)		D1-BS-CDS-R1Seq	RO–AAACACTAACAATCCCTCCAATTAC
*dmrt1*		DMRT1-F	GTGACTCTGACTGGCCCAGAG
(RT-qPCR, Ravi et al. [23])		DMRT1-R	CAGCAGGTCGGACGTTCC
*dmrt1*		DMRT1_Male_F	TGTCTTTTTACTCTCCCTGC
(male-specific RT-PCR)		DMRT1_Male_R	TGGTATTGCTGATAGTTGTAG
*cyp19a1*	KR492506.1	CYP19-BS-F.Seq	FO–TGGTTGTTTATAAAGGGGAAGTTT
(BSAS)		CYP19-BS-R.Seq	RO–CCAACAACAAACAAACAAATAACATA
*cyp19a1*		CYP19a_qPCR_F3	CACTGTTGTAGGTGAGAGACA
(RT-qPCR)		CYP19a_qPCR_R3	CTGTAGCCGTCTATGATGTCA
*cyp19a1*		CYP19a_Female_F	GGTTGTTGTAAATCCTCATCCC
(female-specific RT-PCR)		CYP19a_Female_R1	TCTTATCTGTGTGACTCCAGG
*ubq*	XM_018704769	Lc_ubq_F	ACGCACACTGTCTGACTAC
(RT-qPCR, De Santis et al. [29])		Lc_ubq_R	TGTCGCAGTTGTATTTCTGG
*foxl2 (1)*	KR492507.1	F2-BS-P-F1.Seq	FO–AAAGGGTTGGGTTTATTGATTTATAA
(BSAS)		F2-BS-P-R1.Seq	RO–ATCCAAATACCAACAAACAAAACTT
*foxl2 (2)*		F2-BS-CDS-F1.Seq	FO–AGTTTGTGAGGATATGTTTGAGAAG
(BSAS)		F2-BS-CDS-R1.Seq	RO–CCATACTCTACACCCTAAAATAAAAATTAT
*nr5a2 (1)*	KR492512.1	sf1-BS-F1.Seq	FO–TTTTGTGTGTTTTTATTTGTTTGTG
(BSAS)		sf1-BS-R1.Seq	RO–TTCTTTCTCAATTCTTTTAAACTTTTAAAT
*nr5a2 (2)*		sf1-BS-F2.Seq	FO–GGAAAAGAGATTGTTTAGTATAGTAATAGA
(BSAS)		sf1-BS-R2.Seq	RO–TAAAAACACTAACCTTACAACTCTC
*Amh*	KR492510.1	amh-F.Seq	FO–TGGTGTGTGTTTGAATTAGAAAATT
(BSAS)		amh-R.Seq	RO–CCATAAAAAACATAAAAAACCACAC
*sox8 (1)*	KR492511.1	S8-BS-P*-F2.Seq	FO–TAAATAGGGAAGTAGAAGGGAAATAA
(BSAS)		S8-BS-R.Seq	RO–AAATCCAATTTCTTACCCAAACC
*sox8 (2)*		S8-BS-CDS-F1.Seq	FO–GTTTGGGTAAGAAATTGGATTT
(BSAS)		S8-BS-R2.Seq	RO–TAACTACTCTATTATTTTCATTTAATACAA
*sox9*	KR492508.1	S9-BS-F2.Seq	FO–ATTTAGTTTTGTTAGTTAAGTTGTG
(BSAS)		S9-BS-R2.Seq	RO–TACAAACAAAAAACTTTTCTTCTTC

^**1**^ FO (5’ TCGTCGGCAGCGTCAGATGTGTATAAGAGACAG) and RO (5’ GTCTCGTGGGCTCGGAGATGTGTATAAGAGACAG) are Illumina’s forward overhang (FO) and reverse overhang (RO) adapter sequences added to locus-specific primer sequences.

Genomic DNA was extracted from testicular and ovarian tissue (~3 mm^3^) using a CTAB-chloroform method and quality and quantity checked on a 0.8% agarose gel and Nanodrop (Thermofisher), respectively. DNA (2 μg) was bisulfite converted using an EZ DNA Methylation^™^ Kit (Zymo Research) and targeted gene amplification was performed with 1 μl of PCR buffer, 0.2 μl of 50 mM MgCl_2_, 0.3 μl of 10 mM dNTPs, 0.2 μl of forward and reverse primers, 0.04 μl of Platinum Taq DNA polymerase (Thermofisher), 7.26 μl of water, 0.8 μl of template in 10 μl PCR reactions using a C1000 (Biorad) thermocycler (95 °C for 2 min, 44 cycles of 95 °C for 30 s, 58 °C for 35 s, 72 °C for 40 s, 72 °C for 10 min). PCR amplicons (2 μl) were checked on a 1.5% agarose gel and the concentration estimated against the Easy Ladder I 250 bp band (Bioline) using ImageJ [30] For each sample, equimolar quantities of PCR products were pooled into small and large amplicons (320 bp cut-off), cleaned with SeraMag Beads (GE Healthcare) following the manufacturer’s protocol. A second round of PCR (reduced to 20 cycles) was then performed to index samples for sequencing with the Nextera XT Index Kit (Illumina) (2.5 μl of 10x PCR buffer, 0.75 μl of MgCl_2_, 0.5 μl of 10 mM dNTPs, 2.5 μl of each N7XX and S5XX indexing primers, 0.1 μl of Platinum Taq DNA polymerase, 14.15 μl of water, 2 μl of PCR template). Indexed samples were cleaned, gel checked and pooled as above for next-generation sequencing (Illumina MiSeq, V3 kit, 300 bp paired-end). Raw reads were quality trimmed (base pair < 95% call accuracy trimmed from end) and filtered (any read < 100 bp removed 100 bp) prior to being mapped to a 4,717 bp *in-silico* bisulfite converted reference sequence containing the 11 targeted regions interspaced by 100 Ns in Geneious (Biomatters) using Bowtie2 [31]. The methylation status within each CpG motif was then calculated by the percentage of unconverted cytosine using Geneious (Biomatters) SNP/variant finder. Kruskal-Wallis analyses were used to assess differential methylation status of each CpG site among testes and ovaries of wild and captive barramundi as homoscedasticity was violated precluding a parametric approach. Differences were considered significant at *P* < 0.05.

To assess the expression levels among individuals of each sex, a complementary RT-qPCR approach targeting *cypa19a1* and *dmrt1* was also performed. Intron-spanning primers for *L*. *calcarifer cyp19a1* were designed with PerlPrimer v1.1.2.1 [32], whereas primers for *dmrt1* and *ubq* (internal reference gene) were derived from Ravi et al. [23] and De Santis et al. [29], respectively (Table 1). *Cyp19a1* and *dmrt1* RT-qPCR primers generated amplicon products for both male and female gonads. Here, gonadal samples were subject to RNA extraction using a NucleoSpin RNA XS Kit (Macherey-Nagel) and residual genomic DNA removed with a Turbo DNA-free Kit (Ambion). RNA quality and quantity where checked on a Nanodrop (Thermo-scientific) and agarose gels, and a subsample further checked on a Qsep100 Analyzer (Precision Biosystems). First strand cDNA was synthesized from 2 μg of RNA using a 1:1 combination of oligo(dT) and random hexamers primers using Tetro cDNA Synthesis Kit (Bioline) and then 1:5 diluted with UltraPure DNase/RNase-Free Distilled Water (Invitrogen). RT-qPCR was carried out in triplicate 15 μl reactions (7.5 μl of 2x SsoFast EvaGreen Supermix (Biorad), 0.6 μl of forward and reverse primers (10 mM), 1.3 μl of water, 5 μl of cDNA) in a RotorGene thermocycler (Qiagen) using the following conditions: 95 °C for 30 s, 40 cycles of 95 °C for 5s and 58 °C (*dmrt1* and *cyp19a1*) or 61 °C (*ubq*) for 15 s, followed by a melt curve analysis (65 °C to 95 °C in 0.5 °C increments) for quality control. RT-qPCR efficiencies for each gene were validated using standard curves from five point serially diluted (1:10) cDNA samples (0.98~1.06, R^2^ > 0.99). In addition, a subset of RT-qPCR products for each gene was size checked on a 1.5% agarose gel and confirmed through Sanger sequencing (Australian Genomic Research Facility). *Dmrt1* and *cyp19a1* expression were normalized against that of *ubq* by the 2^-ΔCt^ method of Livak and Schmittgen [19]. Data was log-transformed to conform to normality and equality of variances (Kolmogorov-Smirnov and Levene tests, respectively, *P* > 0.05) and t-tests used to compare the relative expression of each targeted gene between testes and ovaries using the SPSS Statistics 23 software package (IBM). Results were significant at *P* < 0.05. For the remaining *nr5a2*, *amh*, *foxl2*, *sox9* genes, comparative expression levels between *L*. *calcarifer* testes and ovaries were based on previously published RT-qPCR data [23].

In addition, two end-point RT-PCR assays (i.e. on gonadal cDNA) to target *L*. *calcarifer* sex-specific splicing variants of *dmrt1* and *cyp19a1* were designed. The male-specific assay targeted the *dmrt1* DM domain were found to be expressed in testes only, whereas the female-specific assay targeted the 3’-end of *cyp19a1a* exon1, found to be expressed in ovaries. PCR reactions (5 μl of 2x Type-it PCR Buffer (Qiagen), 0.2 μl of forward and reverse primers (10 mM, Table 1), 3.6 μl of water, 1 μl of cDNA template), were carried out in a C1000 Thermal Cycler (Bio-Rad) using the following cycling conditions: 95 °C for 5 min, 8 cycles of 95 °C for 30 s, 57 °C for 90 s and 72 °C for 30 s, then 17 cycles of 95 °C for 30 s, 55 °C for 90 s and 72 °C for 30 s, then a final step of 60 °C for 30 min. PCR amplification was checked by visualisation on a 1.5% TBE agarose gel containing GelGreen (Biotium Inc.), where 2 μl of PCR products were loaded and electrophoresed for 25 min at 80 V and 400 mA.

### Histology

To validate sex status of wild fish, testis and ovaries were cut into 1 cm long pieces and fixed in 10% neutral buffer formalin for 24 h, then subjected to standard histology procedures. The paraffin embedded preparations were sectioned serially at a 5 μm thickness, mounted on slides and stained with hematoxylin—eosin (H&E) and examined with Olympus microscope and cellSens Digital Camera System (Olympus, Japan).

## Results

**Fig 1:**
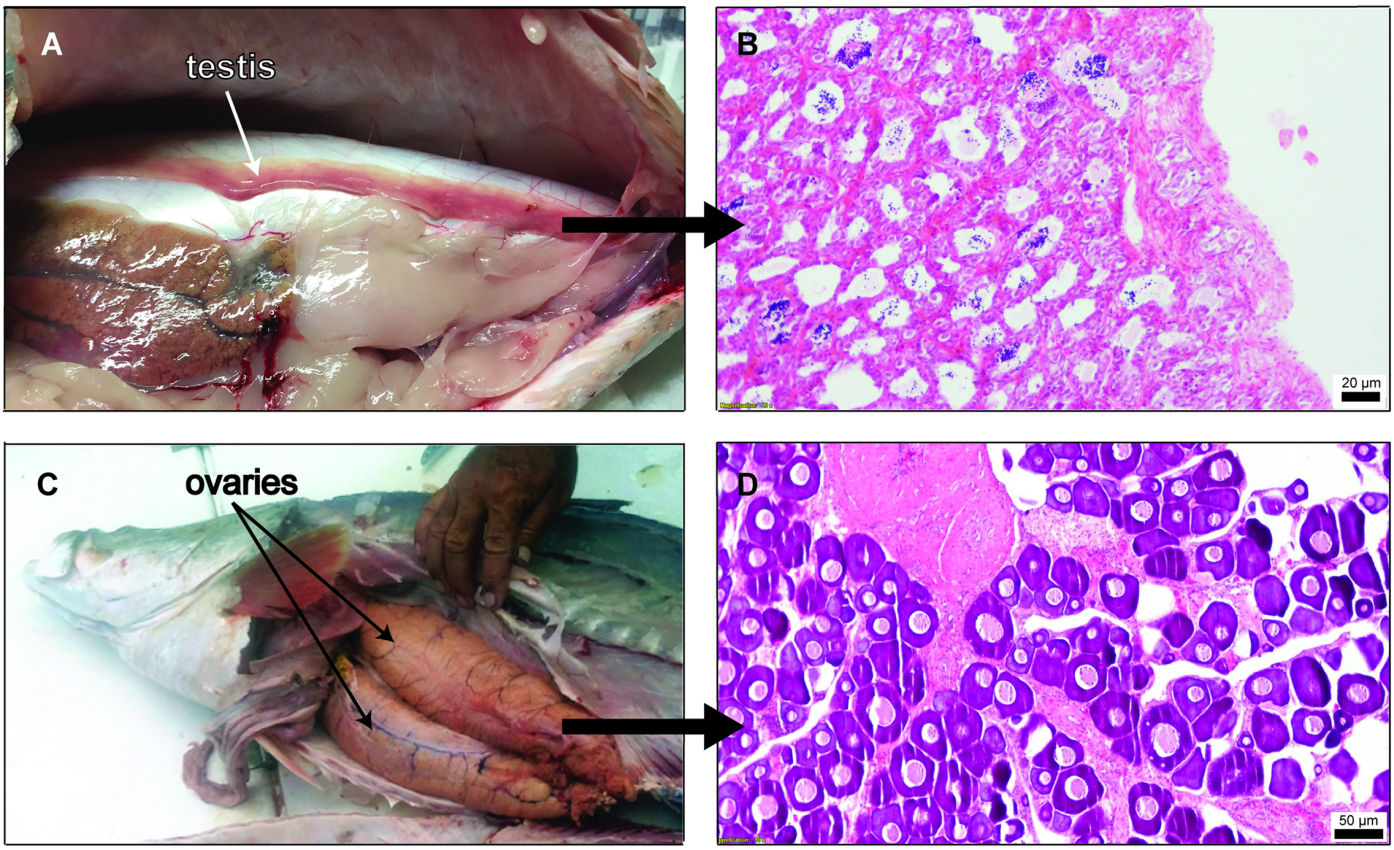
Macro and H&E histology photographs of testis (a) and (b) and ovaries (c) and (d) of representative wild-caught adult *Lates calcarifer* individuals used in the study, showing the significant morphological and cellular transformations which have to occur during the natural male-to-female sex change.

Gonads of adult *L*. *calcarifer* sampled in this study were assessed to confirm sex and maturation stage. In captive broodstock, motile spermatozoa in testes (n = 14) and vitellogenic oocytes (ø = 150–450 μm) in ovaries (n = 14) obtained through cannulation biopsies indicated that individuals were in late maturation stages. In wild caught fish, three gonads of each sex were analyzed through histology. Spermatozoa were present within the lumen of the seminiferous tubules of the barramundi testes, whereas in female barramundi, eggs were present in pre-vitellogenic and vitellogenic stages. Overall, adult individuals were in late gonadal maturation (Fig 1).

### DNA methylation levels between captive and wild males and females

A total of ~3.5 million clean reads from 42 *L*. *calcarifer* adults were mapped against the *in-silico* bisulfite converted reference sequence of *dmrt1*, *cyp19a1*, *sox8*, *sox9*, *foxl2*, *nr5a2* and *amh*, yielding a mean coverage of ~7,000 reads per amplicon (Table 2).

**Table 2:** Number and weight of adult *Lates calcarifer* individuals and number of clean reads from bisulfite amplicon next-generation sequencing mapped against the seven sex-related genes sequences.

Group	n	Fish weight range; mean ± S.D. (Kg)	Clean reads mapped
Male Broodstock	14	5.46 ~ 8.19; 6.92 ± 1.11	1,111,247
Male Wild	10	2.35 ~ 8.99; 5.39 ± 2.54	1,068,190
Female Broodstock	14	7.34 ~ 12.50; 9.97 ± 1.33	1,137,133
Female Wild	4	9.29 ~ 16.68; 12.93 ± 3.12	179,848

**Fig 2:**
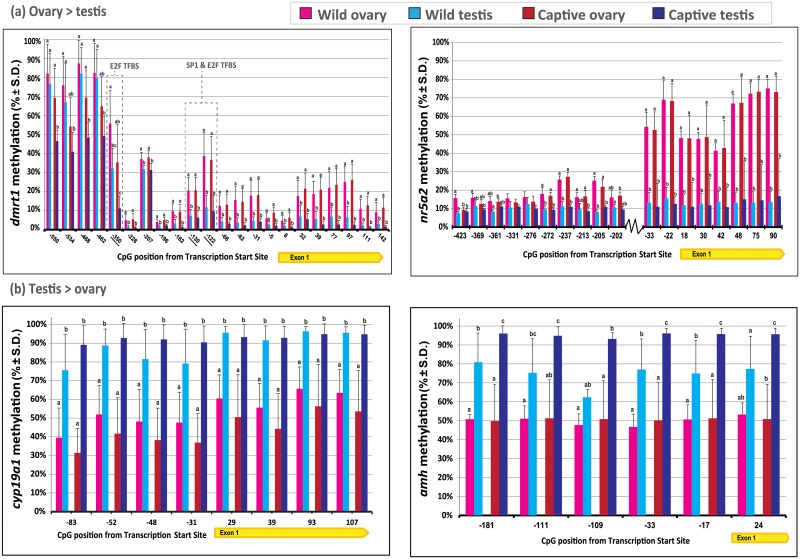
DNA methylation profiles (mean % ± S.D.) of barramundi *Lates calcarifer* sex-related genes within testes (n = 10) and ovaries (n = 4) of wild caught individuals and testes (n = 14) and ovaries (n = 14) of captive broodstock, as obtained by bisulfite amplicon next-generation sequencing. (a) female-biased methylated genes *dmrt1* and *nr5a2*; (b) male-biased methylated genes *cyp19a1* and *amh*. Within each CpG site, different letters denote significant differences between groups (*P* < 0.05).

Four out of the seven genes investigated in this study presented distinctive sex specific DNA methylation patterns (Table 3). Barramundi *dmrt1* and *nr5a2* had higher levels of methylation in ovaries than in testes (*P* <0.05), where the greatest differences were observed within 5’-UTR and first exons, rather than in distal promoters (Fig 2a). Most notably, *nr5a2*, which encodes the liver receptor homolog 1 (LRH1) protein, showed the most distinctive differences for epigenetic markers between sexes, with a methylation average difference of 46.2% and up to 58.9% in CpGs +75 and +90. In addition, *dmrt1* and *nr5a2* genes presented a highly variable methylation pattern across individual CpG sites (Fig 2a). Although average ovarian *dmrt1* methylation was only 10.1% greater than that of testes for the 17 CpG sites investigated, two CpG sites located in the proximal promoter differed by greater than 20% (-350 bp and -122 bp). These CpG sites corresponded to putative transcription factor binding sites E2F and SP1 (Fig 2a). CpG methylation levels were consistent among individuals of the same sex, regardless of their origin and sampling procedure (i.e. dissection on wild animals *vs*. cannulation biopsy on captive broodstock). In contrast, *cyp19a1* (average of 41.2%, up to 46.8% in CpG -83) and *amh* (average of 32.7%, up to 37.3% in CpG +24) were more methylated in testes than in ovaries (Fig 2b), where similar methylation levels were observed among distinct CpG sites, although variable levels of methylation were evident depending on the origin of fish (captive *vs*. wild). Although a number of putative transcription factor binding sites were identified within the 1,400 bp region upstream of *cyp19a1* CDS (e.g. 20 Sox/testis determining and related HMG box factors, five forkhead domain factors, five estrogen related receptors, two cAMP, one DMRT3, one DMRT5, one SF1 and one LHR), the nearest putative TFBS to a *cyp19a1* CpG site investigated in this study was at least 41 bp away (a cAMP upstream of CpG -83). Of note, *L*. *calcarifer cyp19a1* has a very low CpG density in its promoter, possessing only 7 CpG sites in the 1,400 bp region upstream of its start codon (i.e. one CpG per 200 bp). Of all genes investigated in this study, *amh* was the only gene where significant differences in methylation levels were observed among the four groups (captive male > wild male > captive female > wild female) (Table 3; Fig 2b). Lastly, *foxl2*, s*ox8* and *sox9* were mostly hypomethylated (<10% methylation) in both testicular and ovarian tissues, with overall lowest levels detected in captive males (Table 3, S1 Fig).

**Table 3:** Differential methylation patterns (mean percentage ± S.D.) within sex related genes targeted by bisulfite amplicon next-generation sequencing among *Lates calcarifer* adults of captive and wild origin. Different letters denote significant differences among groups (*P* < 0.01).

Gene (amplicon #)	Amplicon size (position from TSS)	# CpG Per amplicon	Testes (%)		Ovaries (%)		Overall (%)
			Captive	Wild	Captive	Wild	
*dmrt1 (1)*	270 bp (-575, -305)	5	41.2 ± 17.1 ^a^	67.8 ± 20.2 ^ab^	58.6 ± 14.6 ^ab^	71.0 ± 12.3 ^b^	59.6 ± 19.1
*dmrt1 (2)*	428 bp (-255, +173)	17	6.8 ± 6.9 ^a^	6.5 ± 7.3 ^a^	17.0 ± 9.5 ^b^	16.3 ± 10.4 ^b^	11.6 ± 9.8
*nr5a2 (1)*	302 bp (-450, -148)	10	10.2 ± 1.3 ^a^	10.7 ± 1.4 ^ac^	16.2 ± 4.8 ^bc^	20.6 ± 4.4 ^b^	14.4 ± 5.4
*nr5a2 (2)*	194 bp (-79, +115)	8	12.9 ± 2.2 ^a^	13.4 ± 1.1 ^a^	59.3 ± 12.4 ^b^	59.3 ± 12.9 ^b^	36.2 ± 24.9
*cyp19a1*	267 bp (-107, +160)	8	92.5 ± 2.0 ^a^	88.0 ± 8.2 ^a^	44.1 ± 8.7 ^b^	54.0 ± 8.9 ^b^	69.6 ± 22.4
*Amh*	341 bp (-258, +83)	6	94.0 ± 1.4 ^a^	76.6 ± 3.0 ^b^	59.5 ± 2.2 ^c^	46.0 ± 2.7 ^d^	69.0 ± 18.5
*foxl2 (1)*	255 bp (-282, -27)	9	3.7 ± 0.7 ^a^	6.9 ± 0.6 ^b^	5.8 ± 0.8 ^bc^	4.0 ± 0.7 ^ac^	5.1 ± 1.5
*foxl2 (2)*	308 bp (+374, +682)	18	6.4 ± 0.9 ^a^	8.61 ± 0.9 ^b^	7.55 ± 1.1 ^ac^	8.5 ± 1.0 ^bc^	7.8 ± 1.3
*sox8 (1)*	368 bp(-195, +173)	26	1.5 ± 1.2	2.0 ± 1.0	2.3 ± 1.3	2.4 ± 2.6	2.0 ± 1.7
*sox8 (2)*	353 bp (+151, +504)	20	7.1 ± 1.2 ^a^	10.0 ± 1.7 ^b^	9.1 ± 1.5 ^b^	10.5 ± 2.5 ^b^	9.2 ± 2.2
*sox9*	344 bp (-130, +214)	19	0.8 ± 0.8	1.1 ± 0.9	1.1 ± 0.7	1.3 ± 0.7	1.1 ± 0.8

### *Dmrt1* and *cyp19a1* expression levels and sex-specific alternative splicing

**Fig 3:**
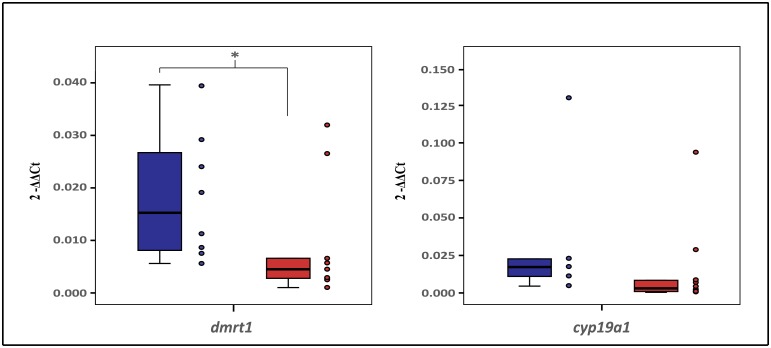
Expression levels of *dmrt1* and *cyp19a1* from testes and ovaries of barramundi *Lates calcarifer* (n = 5–10 per sex/gene). * denotes significant differences between gender (*P* < 0.01).

**Fig 4:**
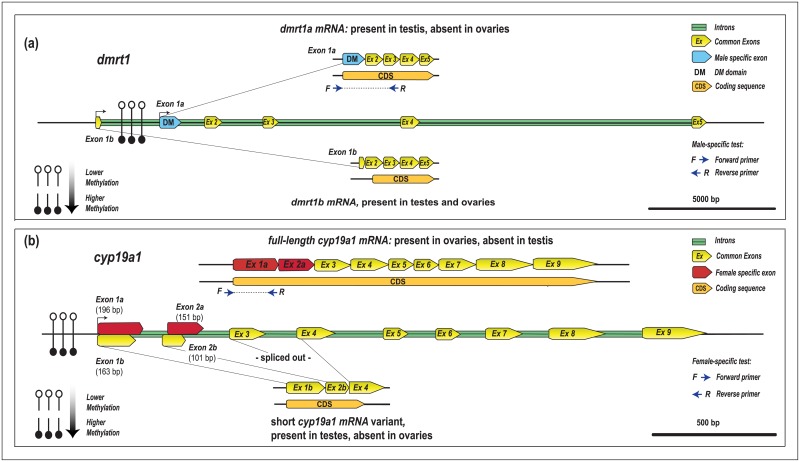
Promoter DNA methylation and mRNA alternative splice variants of (a) *dmrt1* and (b) *cyp19a1* in gonads of the protandrous hermaphrodite barramundi *Lates calcarifer*.

Results from RT-qPCR revealed that *dmrt1* expression was on average 1.9 times higher in the testis than in ovaries, whereas no sex-specific differences were found for *cyp19a1* (Fig 3). Further investigation using a publicly available [33] *L*. *calcarifer* RNASeq library of one testes and one ovary (NCBI SRA accessions SRX867251 and SRX867252), whereby raw reads were mapped back to *L*. *calcarifer dmrt1* and *cyp19a1* genes (NCBI Ref. KR232516.1 and KR492506.1, respectively) suggested that both *dmrt1* and *cyp19a1* were alternatively spliced in a sex-specific manner. Molecular cloning and Sanger sequencing of *dmrt1* and *cyp19a1*, using cDNA from gonads of adult fish, confirmed shotgun transcriptome findings in that barramundi ovaries lacked the previously known *dmrt1* exon 1 (hereafter named as *dmrt1* exon 1a), which harbours the DM domain. This led to the discovery of an alternative untranslated *dmrt1* exon 1 (59 bp, named *dmrt1* exon 1b) located 3,357 bp upstream of the *dmrt1* start codon. *Dmrt1* exon 1a was transcribed only in testis, while the second variant, *dmrt1b*, was transcribed in both ovary and testes (Fig 4a). In an opposite fashion to *dmrt1* in females, barramundi testes lacked the full-length gonadal aromatase *cyp19a1* mRNA, making instead use of two distinct shorter exons 1 and 2 within the existing CDS (protein coding sequence). In testes, *cyp19a1* exon 1 was shortened from 196 bp to 163 bp, exon 2 was also shortened from 151 bp to 101 bp (i.e. partial intronization of full-length *cyp19a1* exons 1 and 2), and exon 3 was ocasionally spliced out (not observed in ovarian samples) (Fig 4b). Both alternatively spliced forms were submitted to Genbank (*dmrt1b* accession number MH784536, and *cyp19A1* short variant accession number MH784537).

**Fig 5:**
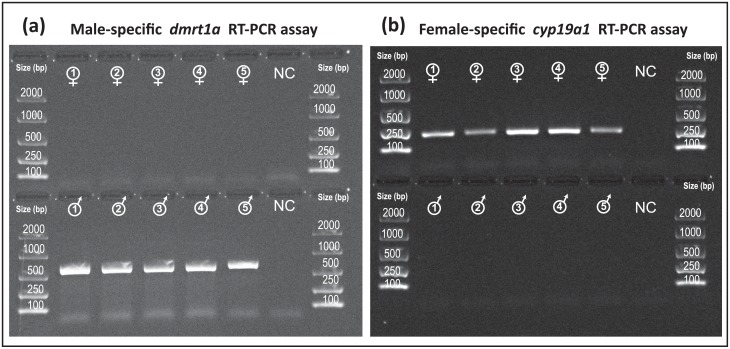
Agarose gel electrophoresis of RT-PCR assays (on cDNA) targeting *dmrt1* male-specific DM domain (a) and *cyp19a1a* female-specific exon 1a (b) in ovaries (top rows, n = 5) and testis (bottom rows, n = 5), with no amplification in gonads of the opposite sex and negative controls (NC). Expected amplicon sizes of 575 bp for *dmrt1* and 207 bp for *cyp19a1a L*. *calcarifer* sex-specific tests.

To confirm barramundi *dmrt1* and *cyp19a1* sex-specific alternative splicing, two sex-specific tests on cDNA of gonads of this protandrous hermaphrodite were developed and validated. Firstly, a male-specific RT-PCR assay targeting the *L*. *calcarifer dmrt1* DM domain was developed, whereby a forward primer positioned immediately upstream of *dmrt1* start codon (i.e. between -24 bp and -3 bp away from *dmrt1* exon 1a) amplified a 575 bp product only in testes, but not in ovarian tissue (Fig 5a). Secondly, a female-specific RT-PCR assay targeting ovarian specific *L*. *calcarifer cyp19a1*, based on a reverse primer positioned at the 3’-end of *cyp19a1* exon 1a (i.e. between +163 bp and +183 bp), amplified a 207 bp product only in ovaries, but not in testes (Fig 5b). It should be noted that *cyp19a1* anomalous shorter variants were not exclusive to testis mRNA. Although all nine expected *cyp19a1* exons (NCBI Ref. KR492506) coding for gonadal aromatase were exclusively found in ovarian samples, multiple alternative spliced transcript variants, frequently presenting exonization of *L*. *calcarifer cyp19a1* gene intronic sequences (most frequently containing introns 3 and 2) were also present in mRNA within gonads of both sexes.

### Relationship between DNA methylation, expression levels and CpG density of sex genes between males and females

As assessed by RT-qPCR targeting transcripts expressed in both sexes of *L*. *calcarifer* adults, no clear patterns in the relationship between DNA methylation and gene expression levels between testes and ovaries were identified for the sex genes investigated in this study. Whereas an inverse relationship between methylation and expression levels was identified for *dmrt1* (10.1% lower methylation and 1.9-fold higher expression in testes vs. ovaries), it was not found for *cyp19a1* (41.2% higher methylation in testes vs. ovaries, and no difference in expression between sexes) (Table 4). Complementing our current analyses with recently published RT-qPCR data expression levels of sex genes in *L*. *calcarifer* of Asian origin, *cyp19a1* was previously found 5.5 times more expressed in testes than in ovaries [23] (Table 4). For the remaining genes, an inverse relationship between methylation and expression levels was identified for *nr5a2* (24.9% lower methylation and 15.2-fold higher expression in testes vs. ovaries), however, a strong direct relationship between *amh* methylation and expression was also observed (32.6% higher methylation and 19.7-fold higher expression in testes vs. ovaries). Within the hypomethylated genes *foxl2*, *sox9* and sox8 no differences in DNA methylation levels were observed between sexes (*P* > 0.05). In terms of gene expression level, *sox9* was observed to be 51.8-fold more expression in testes vs. ovaries, whereas no differences had been reported for foxl2 (*sox8* expression has not been assessed) (Table 4).

**Table 4:** Overall differences in DNA methylation levels across 146 CpG sites and expression levels of seven sex-related genes between testes and ovaries of *Lates calcarifer* (*P < 0.05, **P < 0.01, *n*.*s*. = no significant differences *P* > 0.05).

Gene	Methylation differences (absolute %)	Fold change in gene expression^(1)^	Fold change in gene expression^(2)^
	Testes vs. Ovaries		
*dmrt1*	-10.1**	7.8**	1.9*
*cyp19a1*	41.2**	5.5**	2.5 (*n*.*s*.*)*
*nr5a2*	-24.9**	15.2**	-
*amh*	32.6**	19.7**	-
*foxl2*	-0.2 (*n*.*s*.)	4.9 (*n*.*s*.)	-
*sox9*	-0.3 (*n*.*s*.)	51.8**	-
*sox8*	-0.9 (*n*.*s*.)	-	-

^(1)^ Ravi et al. [23]

^(2)^ current study.

**Fig 6:**
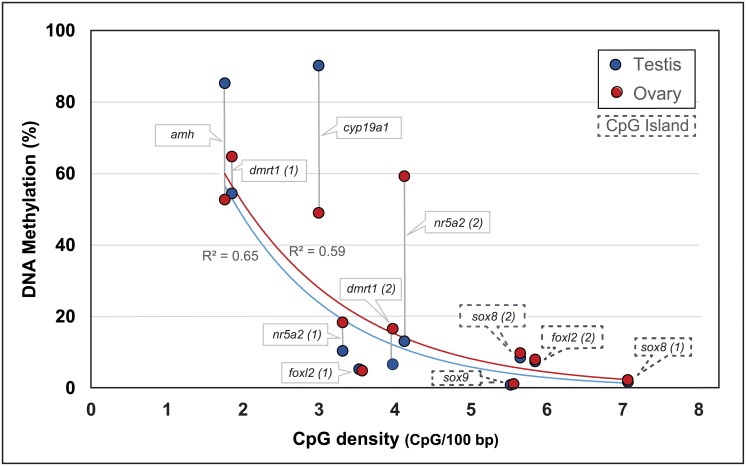
Relationship between DNA methylation and CpG density within proximal promoter and first exon of sex-related genes from testes and ovaries of *Lates calcarifer*. DNA sequences within amplicons in dashed boxes contain atypically high CpG densities known as CpG islands.

Within the 11 amplicons which targeted 146 CpG sites spanning across 3,430 bp of proximal promoters and first exons of seven sex related genes of 24 males and 18 females, an inverse exponential relationship between CpG density and differential methylation levels was observed (Fig 6). Genes which harbored higher CpG densities (also known as CpG Islands) were hypomethylated (*foxl2*, *sox8* and *sox9*) in both sexes. Contrarily, genes harboring CpG sites in low density (~ 4 CpGs per 100 bp or less) had not only higher overall methylation levels but had also greater differences between testis and ovaries (Fig 6).

## Discussion

As assessed by a high-resolution 5-methylcytosine BSAS approach, testes (n = 24) and ovaries (n = 18) of adult *L*. *calcarifer* exhibited significant differences in DNA methylation patterns within the proximal promoter and first exons in *dmrt1*, *cyp19a1*, *amh* and *nr5a2*. Moreover, such methylation patterns were consistently similar within and between sexes, independent of fish origin (farmed broodstock or wild fish) and sampling technique (cannulation of sedated animals or destructive biopsies). This study also revealed two concurrent sex-specific alternative splicing forms within *dmrt1* and *cyp19a1* (gonadal aromatase) genes, genes largely recognized as the two opposing central players in sex determining pathways in hermaphrodites [7, 16, 17], but also in gonochoristic species [1–4, 34]. The DM-domain from *dmrt1* mRNA was completely spliced out in female gonads and contrarily, aromatase exons 1 and 2 were partially spliced out from male gonads, as validated through two novel sex-specific RT-PCR assays. Transitional gonads were not found in 122 individuals sampled, likely due to the rapid transition phase reported from male-to-female [35, 36]. Whether promoter methylation of *dmrt1* (and *nr5a2*) and demethylation of *cyp19a1* (and *amh*) during barramundi sex inversion is a progressive phenomenon like it has previously been reported for the protogynous ricefield eel [16] and for the protandrous black porgy [17], still remains to be elucidated. Nevertheless, this report contributes to the recent body of work aiming to elucidate the genetic mechanisms regulating key reproductive genes involved within the gonads of the large protandrous hermaphrodite barramundi [23, 33] and for the first time provides evidence for epigenetics and alternative splicing affecting genes central to sex differentiation pathways in this important aquaculture species.

Recent research has provided strong links between DNA methylation, gene expression and sex determination in fishes [9], with a particular focus to *cyp19a1*. For a number of species, such as the European seabass [8], black porgy [17], Nile tilapia [15] Japanese flounder [37], ricefield eel [16] and zebrafish [10], higher methylation levels within the testes *cyp19a1* promoter region have been correlated with lower gene expression levels and implied decreases in aromatase production. For those species, hypermethylation of the testicular *cyp19a1* promoter have been associated with gene silencing by regulating the binding of transcription factors (such as SF-1, FOXL2 and CREB) and thereby attributed as a crucial mechanism for establishing sexually dimorphic expression of gonadal aromatase and maintenance of the male gonadal phenotype. However, a female-biased *cyp19a1* transcription abundance was not the case in barramundi, in agreement with a previous study [23]. As assessed by RT-qPCR targeting barramundi *cyp19a1* transcripts expressed in both sexes, similar transcriptional levels between males and females were found in this study, regardless of testicular hypermethylation (*cyp19a1* promoter > 90% methylated in males). In a previous study on Singaporean *L*. *calcarifer* samples which used a different set of *cyp19a1* primers, aromatase was found to be up-regulated in testis [23]. The present study elucidates that although transcription of the *cyp19a1* gene in barramundi can still be detected in testes in levels similar to, or higher than those found in ovaries, notably the full aromatase coding sequence is absent in the males due to exon splicing. In the medaka (*Oryzias latipes*), XX mutants with premature stop codons in *cyp19a1* underwent ovary degeneration, followed by testicular tissue formation [38]. Similarly, TALEN and/or CRISPR/Cas9 mediated *cyp19a1* mutations have been shown to cause higher *dmrt1* expression and partial sex reversal in XX (genetically female) tilapia [39] and all-male offspring in zebrafish mutants [40]. It is important to note that gonadal *cyp19a1* expression has not been found ubiquitously ovarian-biased in teleosts (as reviewed by Guigen et al. [41]). For instance, in the black porgy high *cyp19a1* transcriptional abundance has been linked with testicular differentiation, but not with early ovarian development [42]. In the Ectodine lineage of East African cichlids, similar *cyp19a1* expression levels were found between testes and ovaries [43] and in the half-smooth tongue-sole [44] *cyp19a1* has been found to be up-regulated in testis. Although spliced isoforms of *cyp19a1* are yet to be reported within male gonads of other teleosts, by using similar methodologies targeting short transcripts (a pre-requisite in RT-qPCR) studies may potentially disregard a role for alternative aromatase isoforms.

Alternative splice variants of *dmrt1* have been identified in other teleosts, such as the ricefield eel [45] and zebrafish [46]. To date, only the catfishes *Clarias gariepinus and C*. *batrachus* [47], and now barramundi, were reported to present a 5’ end splice variant lacking the DM domain. Raghuveer and Senthilkumaran [47] have suggested that *dmrt1* splice variants in catfish may regulate the activity of the main isoform (*dmrt1a*). In contrast to catfish in which none of the three *dmrt1* variants were detected in ovaries, barramundi females only expressed the DM-less variant. Recent studies have linked the alteration of *dmrt1* coding sequences with the interruption of male functionality. For instance, RNAi designed to specifically target the *dmrt1* DM-domain has been shown to suppress transcription and reduce germ cell numbers in the testis and stimulate a male-to-female sex change in the protandrous black-porgy [48]. Sex-biased expression of spliced isoforms have also been observed in other important aquaculture species, such as the European seabass (e.g. female-biased *sb sox17* expression) [49] and again in the protogynous rice field eel (e.g. male biased *cyp17a1* expression) [50], suggesting splicing mechanisms to play a role in fish sex determination. In the context of sex determination in fishes, the link between DNA methylation and alternative splicing was first evidenced in the tongue-sole female-biased *figla* (*factor in the germline alpha*) gene, whereby hypomethylated *figla* in testis recruits an alternative first exon devoid the functional helix-loop-helix DNA binding domain (44). Whilst DNA methylation was originally thought to only affect transcription, emerging evidence shows that the splicing of about 22% of alternative exons is regulated by DNA methylation [51]. In the honeybee *Apis mellifera*, for example, splicing of the anaplastic lymphoma kinase gene (alk, an important regulator of metabolism) is regulated by differential methylation and results in phenotypic plasticity of individuals sharing a common genotype (queens and workers) in order to determine caste [52, 53].

Although the exact molecular regulatory systems inducing alternative splicing are yet to be elucidated in barramundi, it is conceivable that differential DNA methylation levels within the promoter and exonic regions in key sex controlling genes might regulate the recruitment of particular exons in this species in a similar fashion to the *figla* gene in the tongue-sole [44], allowing flexibility in whether active domains are included or not in the resultant transcribed mRNA. In particular, DNA methylation levels of *dmrt1* and *cyp19a1* could regulate the presence or absence of functional transcripts, as observed by the loss of the *dmrt1* DM-domain and gain of the full-length *cyp19a1* CDS in females. However tempting it may seem to attribute the non-existence of the full-length *dmrt1* mRNA in females and *cyp19a1* mRNA in males to the higher DNA methylation levels in the promoters and first exons of these genes, it is important to note that DNA methylation-independent mechanisms regulating barramundi *dmrt1* and *cyp19a1* splicing were not investigated in this study, as splicing variants are not always associated with DNA methylation in promoter regions (and vice versa). Therefore, future studies looking into methylation status, expression levels and splicing patterns after gene knock-down or inhibition of DNA methyltransferases in barramundi gonads would be required to confirm this working hypothesis. For *L*. *calcarifer dmrt1*, the greatest differences in methylation levels between sexes were located at CpGs –130 bp and -122 bp situated amidst putative SP1 and E2F TFBS. This particular *dmrt1* proximal promoter region is suggested here to be a key epigenetic and regulatory transcriptional site for *L*. *calcarifer dmrt1*. SP1 TFBS located within -150 bp relative to the major transcriptional start site has been shown to be a regulator of *dmrt1* expression in rat Sertoli cells [54], whereas E2F has been shown to be an efficient transcriptional activator of *dmrt1* in human cell lines [55]. Further experiments aiming to better understand the role of this particular region in the protandry of barramundi are warranted.

*Amh* is invariably up-regulated in juvenile and adults testes in comparison to ovaries (but see Klüver et al. [56]) and for some teleosts *amh* variants were found to be the major sex determining gene, e.g. *amhrII* [57] and *amhy* [5, 58]. In zebrafish, *amh* has been shown to be an important factor leading to the natural ovary transformation into juvenile testis [59] and a decrease in expression levels to be an early sign of sex change in the protandrous black porgy [60]. The direct association between *amh* male-biased expression and male-biased DNA methylation was previously reported in tongue sole by Shao et al. [44]. Similarly to the tongue-sole, *amh* is also highly expressed in *L*. *calcarifer* testes [23], where *amh* was found to be hypermethylated (i.e. average testicular methylation of CpGs within *amh* promoter and first exon was over 85%). Such contrast to expected inverse relationships between promoter methylation and expression warrants further investigation, and the important *amh* gene would be a sound candidate for future studies. New evidence from large scale epigenomic studies are now challenging the conventional assumption that the predominant function of DNA methylation is to repress gene transcription, as this epigenetic modification also targets the bodies of active genes [61]. In the tongue sole and tilapia, such large scale epigenomic studies focused on the role of DNA methylation within sex determination pathways have shown that differential methylation between male and female gonads was only positively correlated with expression levels in about half of genes investigated [15, 44].

In addition to the seven genes investigated in this study, future comparative transcriptomics research using a larger sample size may unravel differentially spliced genes between male and female gonads and their association with promoter methylation. Targeting such genes will allow for a better understanding of the relationship between DNA methylation, expression and splicing in the context of sex differentiation in barramundi. Here, distinct associations observed between DNA methylation and expression levels relative to CpG density within proximal promoters suggests that the epigenetic regulation of transcriptional activity of sex genes may also operate in a CpG density dependent context. Therefore, although the exact role of DNA methylation upon transcriptional levels and phenotypes is still not entirely understood, future studies may unveil the biological role of CpG-density context upon the epigenetic regulation and its associated importance in sex determining pathways.

## Supporting information

S1 Fig Non-biased hypomethylated sex related-genes. (a) *sox9* and (b) *foxl2* (mean % ± S.D.) of barramundi *Lates calcarifer* within testes (n = 10) and ovaries (n = 4) of wild caught individuals and testes (n = 14) and ovaries (n = 14) of captive broodstock, as obtained by bisulfite amplicon next-generation sequencing.
(PDF)
