# Supplementary material for: Sex-specific dmrt1 and cyp19a1 methylation and alternative splicing in gonads of the protandrous hermaphrodite barramundi
Source: PLoS One. 2018 Sep 18;13(9):e0204182. doi: 10.1371/journal.pone.0204182 (PMC6143260; doi:10.1371/journal.pone.0204182)
Supplement: S1 Fig — (a) sox9 and (b) foxl2 (mean % ± S.D.) of barramundi Lates calcarifer within testes (n = 10) and ovaries (n = 4) of wild caught individuals and testes (n = 14) and ovaries (n = 14) of captive broodstock, as obtained by bisulfite amplicon next-generation sequencing. (PDF) [file pone.0204182.s001.pdf]

Supporting information.

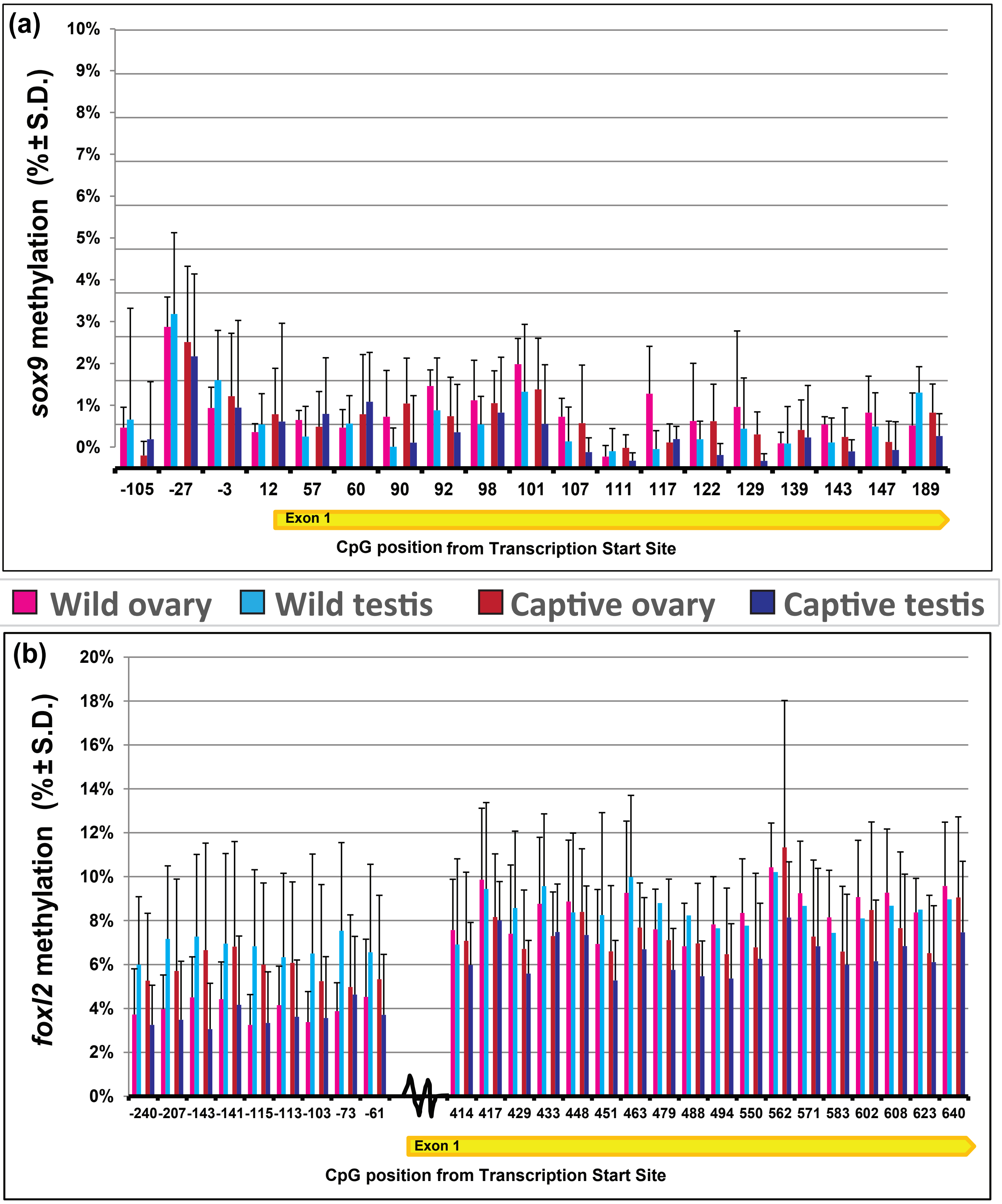

S1 Fig. Non-biased hypomethylated genes (a) *sox9* and (b) *foxl2* (mean % ± S.D.) of barramundi *Lates calcarifer* within testes (n = 10) and ovaries (n = 4) of wild caught individuals and testes (n = 14) and ovaries (n=14) of captive broodstock, as obtained by bisulfite amplicon next-generation sequencing.
